# Enhanced Thermoelectric Properties of Composites Prepared With Poly(3,4-Ethylenedioxythiophene) Poly(Styrenesulfonate) and Vertically Aligned Se Wire

**DOI:** 10.3389/fchem.2021.791155

**Published:** 2022-01-26

**Authors:** In Yea Kim, Dong Won Chun, Sang-Il Kim, Jae-Hong Lim

**Affiliations:** ^1^ Department of Materials Science and Engineering, Gachon University, Seongnam, South Korea; ^2^ Center for Energy Materials Research, Korea Institute of Science and Technology, Seoul, South Korea; ^3^ Department of Materials Science and Engineering, University of Seoul, Seoul, South Korea

**Keywords:** thermoelectric, Se wires array, PEDOT:PSS, galvanic displacement, carrier scattering

## Abstract

Controlling the electronic transport behavior in thermoelectric composites is one of the most promising approaches to enhance their power factor because this enables decoupling of the correlation between the electrical conductivity and Seebeck coefficient. Herein, we show that the unexpected high power factor of the Se nanowire array embedded in poly(3,4-ethylenedioxythiophene)-poly(styrenesulfonate) (PEDOT:PSS) can be achieved by controlling the interfacial band structure engineering. The electrical conductivity and Seebeck coefficient simultaneously increased, confirming that the synthesis of organic/inorganic hybrid thermoelectric materials with improved performance was possible. Our exploration can be helpful for the rational design of high-performance thermoelectric composites through interface engineering.

## Introduction

Considering the expansion of thermoelectric (TE) materials to future energy and electronic devices, functional aspects (e.g., flexibility, transparency, and lightweight) need to be improved to widen their applicability (William and Wong, 2009; Reuss et al., 2015; Oh et al., 2016; Varghese et al., 2016; Ou et al., 2018). Organic TE materials have the potential for application in low-temperature energy harvesting systems and wearable (or flexible) heating and cooling devices (Yue and Xu, 2012; Wang and Yu, 2019). Recently, desirable results regarding the electronic transport properties of poly(3,4-ethylenedioxythiophene) (PEDOT)-based materials (Park et al., 2012; Kang et al., 2016; Kang and Snyder, 2017), such as the high TE performance of PEDOT:poly(styrenesulfonate) (PSS) and PEDOT:poly(3,4-ethylenedioxythiophene)-tosylate), have been demonstrated by engineering the degree of conformation at the molecular level (Bubnova et al., 2011; Kim et al., 2013; Noriega et al., 2013; Cho et al., 2014). An enhanced TE figure of merit (*zT* = *S* ^2^ *σT*/*κ*, where *S*, *σ*, and *κ*, are the Seebeck coefficient, electrical conductivity, and total thermal conductivity at a given absolute temperature *T*, respectively) of 0.42 was achieved at 300 K by mixing dimethyl sulfoxide with commercial PEDOT:PSS (Kim et al., 2013)^13^; however, it remains unsatisfactory for commercial applications. The limited *zT* values in PEDOT-based organic TE materials are mainly due to their poor electronic transport properties, which results in a low power factor (*σS* ^2^) despite their highly desirable low *κ* values (e.g., *κ* ∼0.2 Wm^−1^ K^−1^ for PEDOT:PSS).

A nanocomposite approach by embedding nanoscale conductive fillers into the PEDOT-based matrix has been carried out to increase the *σ* (Du et al., 2012; Wang et al., 2012; Coates et al., 2013; Ju and Kim, 2016); however, improving the power factor is still challenging owing to the strong correlation between *σ* and *S*. For example, graphene was introduced and a high *σ* was obtained because of the aligned PEDOT:PSS chains along the graphene; however, *S* remained at the intrinsic level (Kim et al., 2012). A hybrid of inorganic compounds with a large *S* and highly conductive organic materials is a potential approach for achieving the theoretical maximum power factor because the independent control of *σ* and *S* becomes possible based on the parallel and series models in the composites. Additionally, the generation of a phase boundary between the organic TE matrix and inorganic compounds can trigger the improvement in *S* benefitting from a carrier filtering effect. This is because *S* is related to the energy derivative of the electronic density-of-states (DOS) and the carrier relaxation time through the Mott relationship (See et al., 2010; Yee et al., 2013; Du et al., 2014; Peng et al., 2016). However, there has been no experimental evidence for decoupling the correlation between *σ* and *S*, even in organic TE composites with well-controlled inorganic nanophases.

In the study by Roh et al. (2017), the inorganic Ge_2_Sb_2_Te_5_ (GST) was arranged into a nanowire, and its performance was evaluated by fabricating a composite with PEDOT:PSS. Since this study produced aligned GST through the nanopattern printing method, the height of the GST nanowire was at the nanoscale. Nevertheless, it was confirmed that the decoupling phenomenon of this material increases the *S*, even though *σ* increases. This establishes that a well-arrayed structure can obtain an improved *zT*. However, its nanoscale length of the wire limits its performance improvement.

Various methods have been used to synthesize nanophase inorganic TEs, for example, methods using microwaves (Wang et al., 2017), the hydrothermal synthesis method (Li et al., 2019), and the electrochemical method (Recatala-Gomez et al., 2020). Among these methods, the galvanic displacement reaction (GDR) offers several advantages, including low synthesis cost and easy reaction conditions (Xin et al., 2021). This method is an electrochemical process driven by the redox potential difference between the sacrificial material and solution reacting ions.

In this study, a wire of length at the microscale was formed through the GDR. It is easier to control the length of the wire to increase the height of the inorganic materials *via* this method as compared to the nanoprinting method. In addition, a high *S* of ≥1,000 μV/K at room temperature is desired; therefore, the most attractive material, Se, is used (Kim et al., 2019). However, to use Se as a high-performance TE material, it is necessary to improve its *σ*, which can be achieved by forming a composite structure with the organic PEDOT:PSS.

## Materials and Methods

### Formation of Se Wire Array

The Se wires were formed on a silicon wafer (2 × 2 cm) using GDR. The wafer used in this work was *p*-type, boron-doped, (100)-oriented silicon. The silicon wafer was cleaned with acetone and ethanol. The solution used for the Se deposition is a combination of two chemicals: hydrofluoric acid (HF, J.T Baker, United States) and a saturated solution of selenium oxide (SeO_2_, Sigma Aldrich, United States). The Se nanowire growth was processed in a Teflon vessel for 24 h at 70°C. After completion of the GDR, the synthesized Se wires were carefully rinsed several times with deionized water and ethanol. The Se wire was then dried in a desiccator for 1 day. The prepared wires were transferred to polydimethylsiloxane (PDMS, Sylgard^®^ 184). The PDMS was prepared as a film and hardened at 25°C for 18 h. The silicon wafer on which the Se wires are grown was then placed on top of the prepared PDMS film and pressed with weak pressure. Throughout this process, Se wires were transferred to the PDMS film surface.

### Formation of Se Wire and PEDOT:PSS Composition

**FIGURE 1:**
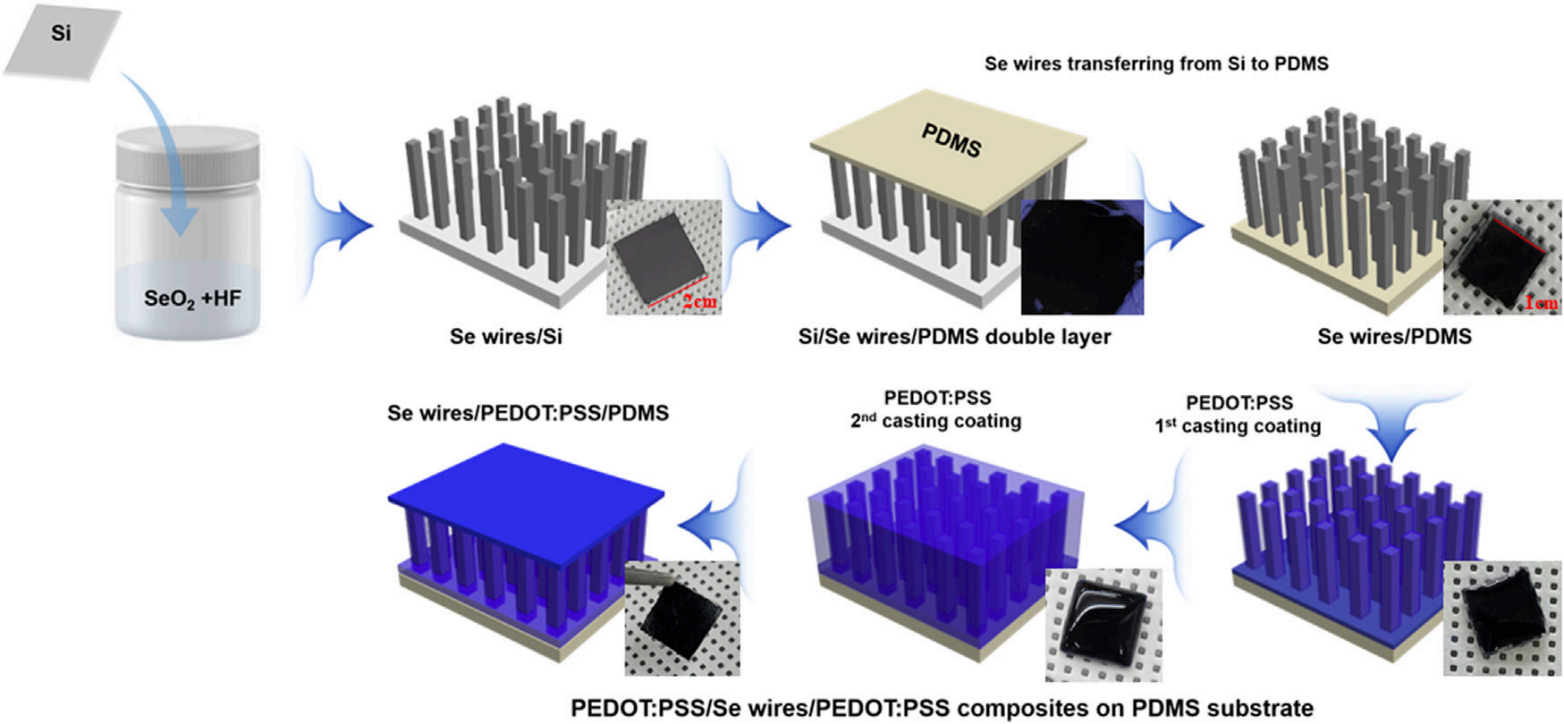
Scheme of the synthesis of the Se wire and poly(3,4-ethylenedioxythiophene)-poly(styrenesulfonate) (PEDOT:PSS) composite.

The effect of doping ethylene glycol (EG, Samchun, Korea) on the TE properties of PEDOT:PSS was determined. According to the volume ratio, 2%, 4%, 6%, 8%, and 10% of EG was added to the PEDOT:PSS solution (Clevios^TM^ PH1000) contained in each vial bottle. Then, the mixed solution of PEDOT:PSS/EG was applied to the Se wire array on the PDMS surface by drop-casting. The Se wire array applied with the PEDOT:PSS/EG mixed solution was dried in a vacuum oven at 40°C for 16 h. In addition, PEDOT:PSS was applied twice to increase the contact between the Se wire and PEDOT:PSS. The process of synthesizing the Se wire and the PEDOT:PSS composite is schematically illustrated in Figure 1.

### Characterization of Prepared Se Wire and PEDOT:PSS

The Se wire array grown *via* the GDR was observed using scanning electron microscopy (SEM, Hitachi S-4200) and scanning transmission electron microscopy energy dispersive spectroscopy (STEM-EDS, Talos F200X, FE, US) at an accelerating voltage of 200 kV (Schottky X-FEG gun) equipped with a Super-X EDS system comprising four windowless silicon drift detectors (SDDs) in the STEM mode with a probe current of ∼0.7 nA. The Se wire and composite structure were measured at 20°–65° (2*θ*) by X-ray diffraction (XRD, Ultima IV). The chemical state of the synthesized Se wire and the compound according to the PEDOT:PSS application was confirmed using X-ray photoelectron spectrometry (XPS, AXIS-NOVA, Kratos Inc.). To measure the work function, ultraviolet photoelectron spectroscopy (UPS, Thermo Fisher Scientific, NEXSA) was used. Conductivity of the synthesized Se wire/PEDOT:PSS composite was measured on a Keithley 2400 Electrometer using the four-point probe technique. The Seebeck coefficient was measured using Seebeck apparatus built in-house. Three samples were analyzed under each condition, and each measurement was repeated five times.

## Results and Discussion

Se wires were synthesized on a silicon substrate by immersing the Si substrate in a mixed solution consisting of 2 mM SeO_2_ and 4.5 M HF at 70°C. The mobility of GDR is caused by the difference in the redox potential between the solid material and the ionic solution used. This technology uses primitive electrochemical phenomena that form the basis of a battery (Kakati et al., 2017)^30^. When a silicon substrate is immersed in an acidic fluoride solution containing only $SeO_{3}^{2-}$ , the silicon atoms on the substrate are galvanically substituted by $SeO_{3}^{2-}$ because of the difference in the redox potential of SiF_6_^2−^/Si^0^ (*E* ^0^ = −1.24 V vs. NHE) and HSeO_3_/Se (*E* ^0^ = 0.74 V vs. NHE), described in the following equations (Jeong et al., 2013; Majidzade et al., 2018; Saha et al., 2020; Tran et al., 2020):

$$\;Si^{0}\left(S\right)+6F^{-}\to SiF_{6}^{2-}\left(aq\right)+4e^{-}E^{0}=-1.24\text{ vs}.\text{ NHE}$$

$$H_{2}SeO_{3}\left(aq\right)+4H^{+}\left(aq\right)+4e^{-}\to S\;e^{0}\left(s\right)+3H_{2}O\;E^{0}=0.74\;\text{vs}.\text{NHE}$$

**FIGURE 2:**
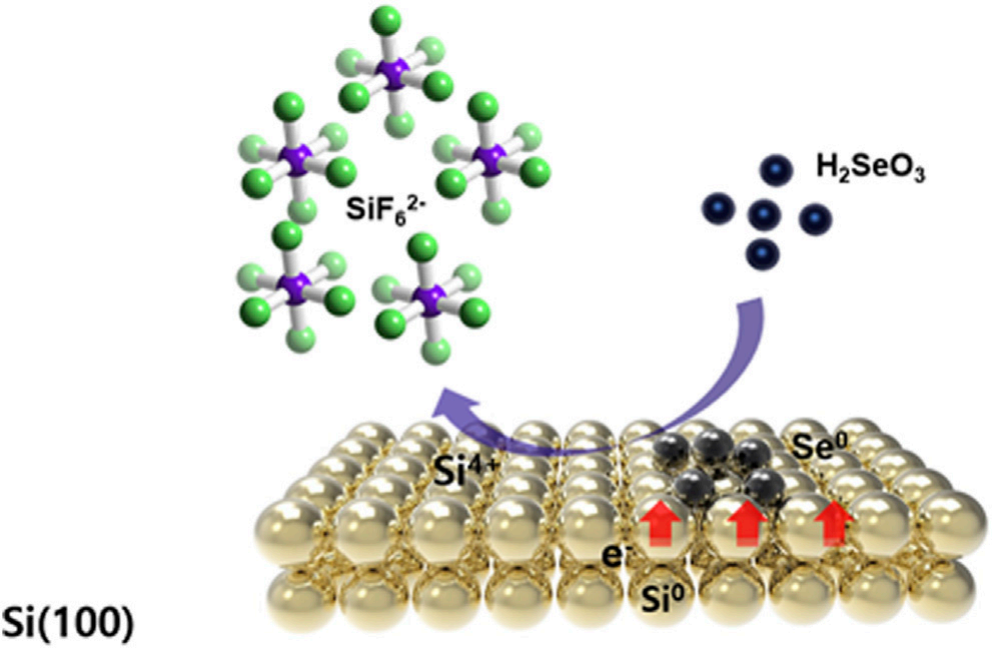
Schematic representation of the synthesis of the Se nanowires by the galvanic displacement reaction of the Si wafer.

During the GDR of Si, positive and negative reactions simultaneously occur on the Si surface as charges are exchanged through the substrate. The fluorine ions in the solution corrode and dissolve the Si substrate in the form of silicon hexafluoride, preventing the formation of passive silicon oxide and helping to maintain the reaction by constantly exposing the new Si surface (Figure 2).

**FIGURE 3:**
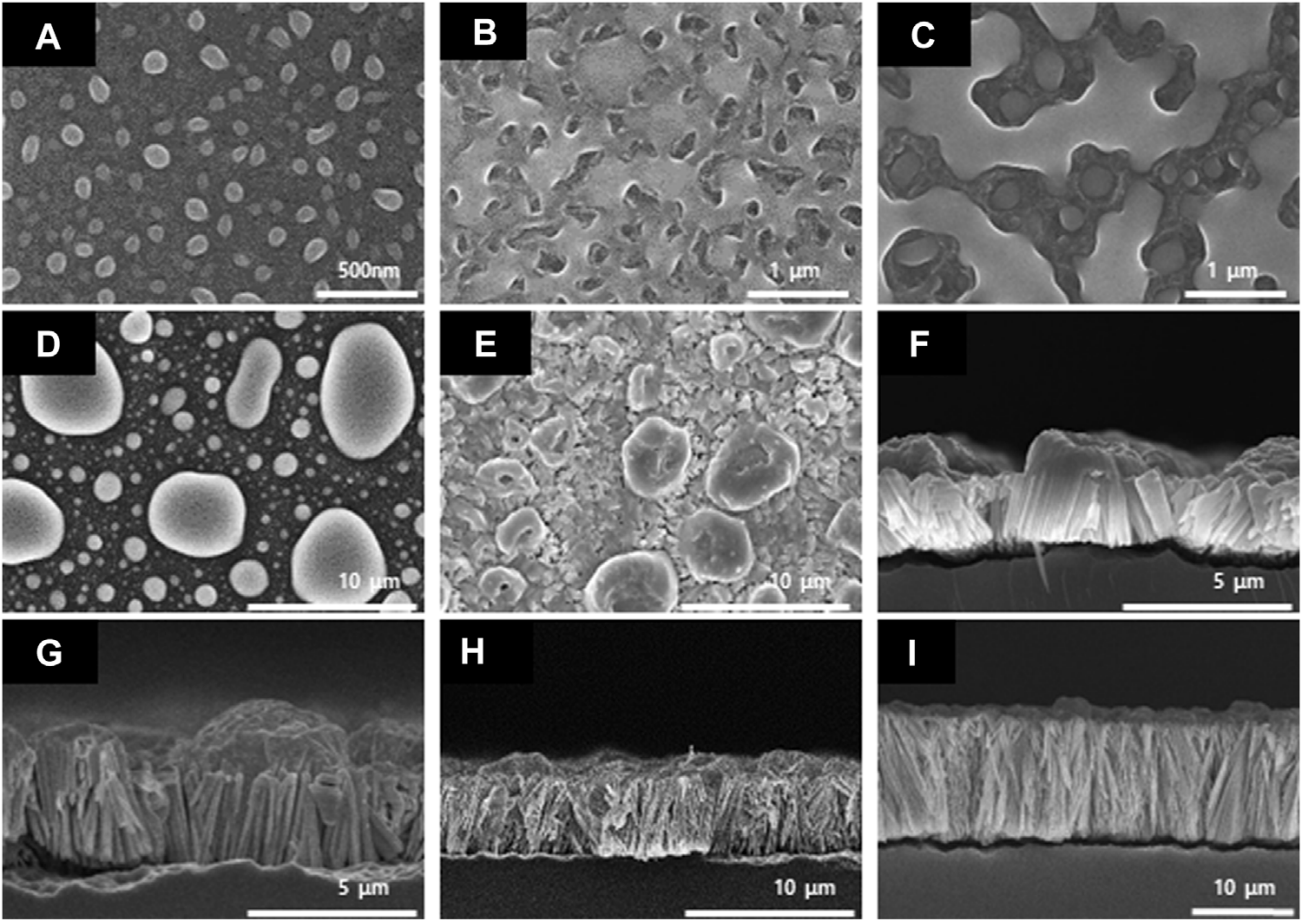
Scanning electron microscopy images of the Se nanowires synthesized by the galvanic displacement reaction at different reaction times. Top-view **(A)** 5 min, **(B)** 30 min, **(C)** 1 h, **(D)** 3 h, and **(E)** 6 h; cross-section **(F)** 6 h, **(G)** 9 h, **(H)** 12 h, and **(I)** 24 h.

As shown in Figure 3, it was confirmed that the nanowires formed by the nucleation and growth mechanisms (Hwang et al., 2019). Furthermore, we were able to determine the optimum reaction time required for the growth of the Se wire through the GDR method on the Si wafer surface. Figure 3A–E demonstrates the initial process of the Se wire formation; the SEM image is shown in the top view. At the initial stage, the 3D Se nuclei were formed on the Si wafer surface (Figure 3A). The mechanism by which this nucleus grows can be described as the Volmer–Weber nucleation and growth mechanism (Choi and Choi, 2013). Initially, the size of the nucleus was 100 nm; as the GDR reaction time increased, the nuclei formed on the surface grew, transforming into an island shape. Then, the grown island-shaped Se coalesced with the adjacent island-shaped Se and completely covered the Si wafer surface in the form of a film (Figure 3E). When the Si surface was completely covered, the reaction was completed on the upper surface. Thus, the reaction between the Si wafer and HSeO_3_^−^ and the growth of the Se wire required more than 6 h. To analyze the length of the Se wire grown *via* the GDR, a cross-section of the sample was obtained after 6 h (Figure 3F); it is apparent that the growth of the wire starts under the Se island. The Se wire grows vertically in the downward direction because a redox potential reaction occurs through Si etching by HF contained in the synthesis solution; however, the upper part of the Si wafer, where the Se thin film is formed, is not etched by HF. Nevertheless, Si is present under the Se thin film, the galvanic reaction occurs because of the etching, and the Se wire starts to grow in a vertical direction (Gadea et al., 2015). The Se wire produced by the 6-h reaction grew to a length of 2.11 μm randomly. However, after 9 h, the Se wire constantly grew in a vertical direction at a growth rate of 390 nm/h.

**FIGURE 4:**
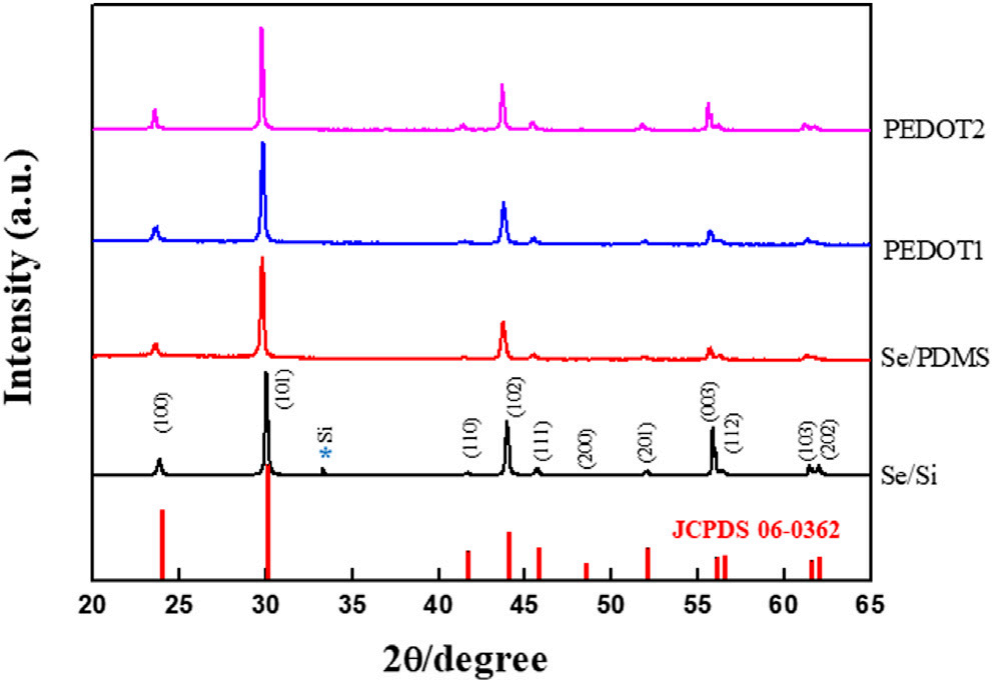
X-ray diffraction pattern of the Se nanowire structure and Se nanowire:PEDOT. The structure change according to the PSS complex process is confirmed.

A composite with PEDOT:PSS with high *σ* and low *κ* was fabricated to improve the TE performance of the Se wire grown by the GDR method. The structure and elemental state of the Se wire and Se wire/PEDOT:PSS were analyzed using XRD and XPS. First, the Se wires were prepared on the Si wafer surface using the GDR method. All reflections of the prepared selenium nanowires are consistent with those of selenium (JCPDS card number 06-0362) (curve a in Figure 4). In addition, the structure of the Se wire, which was transferred to the PDMS, was maintained, and the structural characteristics of the Se wire/PEDOT:PSS composite were confirmed to have similar diffraction patterns to the Se wire. This implies that the composite did not undergo phase separation or change in its crystal structure. However, the reason for the high intensity of (003) in Se/Si is that the Se/Si XRD (003) peak intensity is stronger than other results because of the Se grain size (Lu et al., 1997; Cheng and Samulski, 2003). The reason for the change in the size of the grains is outlined in Figure 3, as the Se nano wire growth direction proceeds downward. Therefore, as the initial Se growth starts from the surface, the grain size is large, hence we were able to obtain this result. In addition, X-ray penetration is difficult owing to the PEDOT:PSS applied to the Se wire surface and transferred to the PDMS.

**FIGURE 5:**
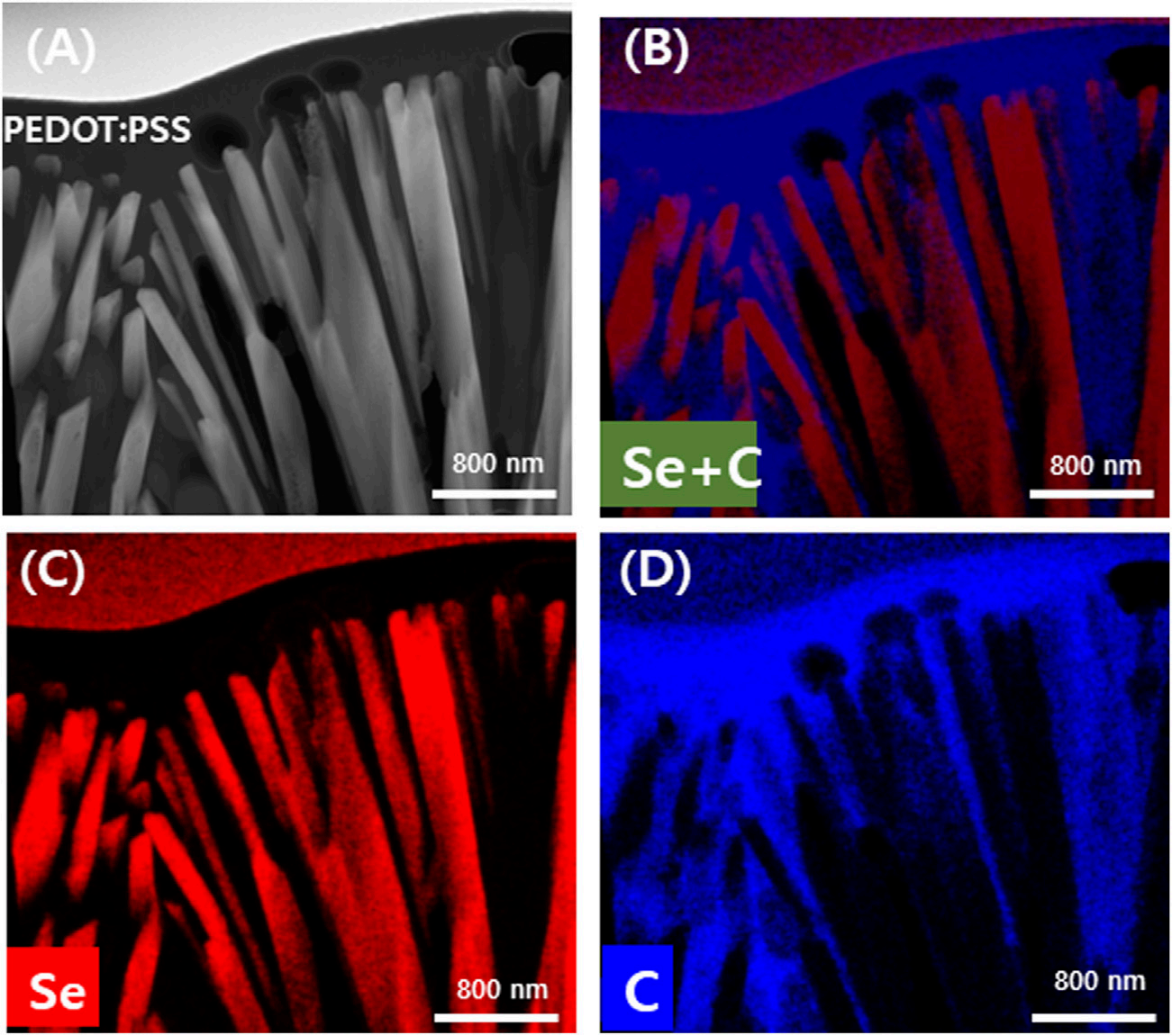
Transmission electron microscopy energy dispersive spectroscopy analysis of the Se nanowires:PEDOT:PSS composite coated twice with PEDOT:PSS. TEM image **(A)** and mapping image according to element **(B)** Se and C **(C)** Se and **(D)** C.

The location of the Se wire and PEDOT:PSS of the manufactured Se wire/composite was confirmed using STEM-EDS. Figure 5A–D shows the STEM and STEM-EDS mapping images of Si wire/PEDOT:PSS. It was revealed that the vertically aligned Se wires were covered by PEDOT:PSS. Figure 5A is a STEM image of a Se wire/PEDOT:PSS composite in which the Se wire and PEDOT:PSS are clearly distinguished. The obtained TEM image was confirmed to be consistent with the images shown in Figure 3I. To clearly distinguish the area identified in the TEM image, specific elements were identified *via* EDS mapping. Figure 5C coincides with the nanowire area composed of Se, and Figure 5D shows the element constituting PEDOT:PSS, which coincides with PEDOT:PSS in Figure 5A. Figure 5B corresponds with the identified Se and C *via* EDS mapping. Additionally, this result can be calculated as the area ratio of the Se wire and PEDOT:PSS. This confirmed that the Se wire and PEDOT:PSS area ratio was 54.57% and 45.43%, respectively. It was clarified that the Se wire surface was sufficiently coated and filled with PEDOT:PSS.

**FIGURE 6:**
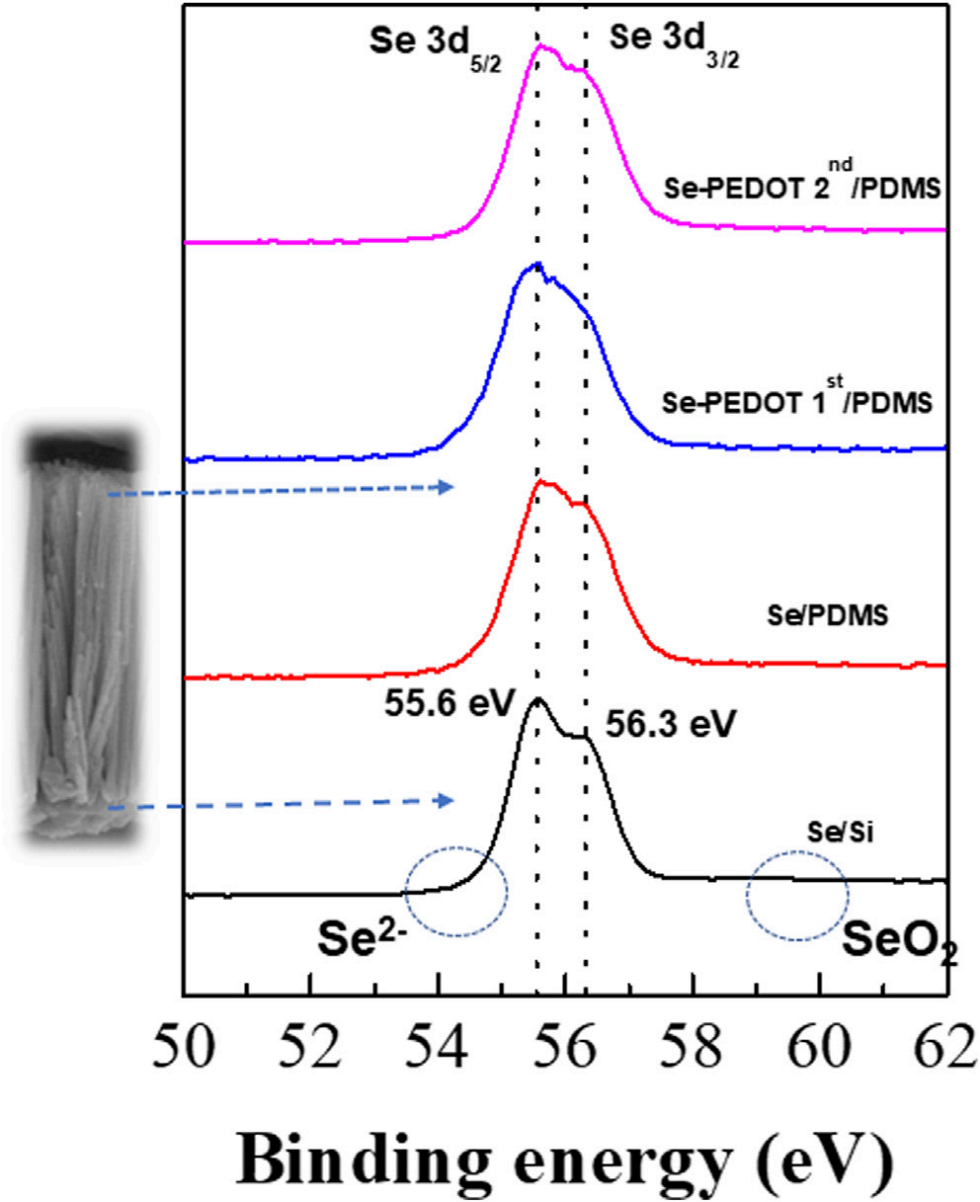
X-ray photoelectron spectra of Se 3d_5/2_ BE and Se 3d_3/2_ of the Se wire:PEDOT:PSS composite.

The interfacial reaction between the Se wires and PEDOT:PSS can be discussed using the XPS spectra of the Se wire/PEDOT:PSS composite according to the first and second applications of PEDOT:PSS (Figure 6). The 3d_3/2_ and 3d_5/2_ binding energies of pure Se appear at 56.3 and 55.4 eV, respectively, and the oxidized Se, Se^2−^ (54.6 eV) or SeO_2_ (59.2 eV), are also observed (Jang et al., 2018). The binding energies of the synthesized Se wire are in the Se_0_ state because 3d_3/2_ and 3d_5/2_ are identified at 55.6 and 56.3 eV, respectively. In addition, no other binding energy was identified as PEDOT:PSS. These results confirm that no oxidation occurred upon applying PEDOT:PSS; therefore, Se remained pure.

The above results also confirmed the viability of synthesizing Se nanowires and Se wire/PEDOT:PSS composites. However, the chain structure of PEDOT:PSS is released as the EG doping content increases (Lee et al., 2016; Roh et al., 2017). This change in the PEDOT:PSS chain structure shows a difference in filling between the Se wires upon application. Therefore, the electrical properties of PEDOT:PSS can be tuned using EG.

**FIGURE 7:**
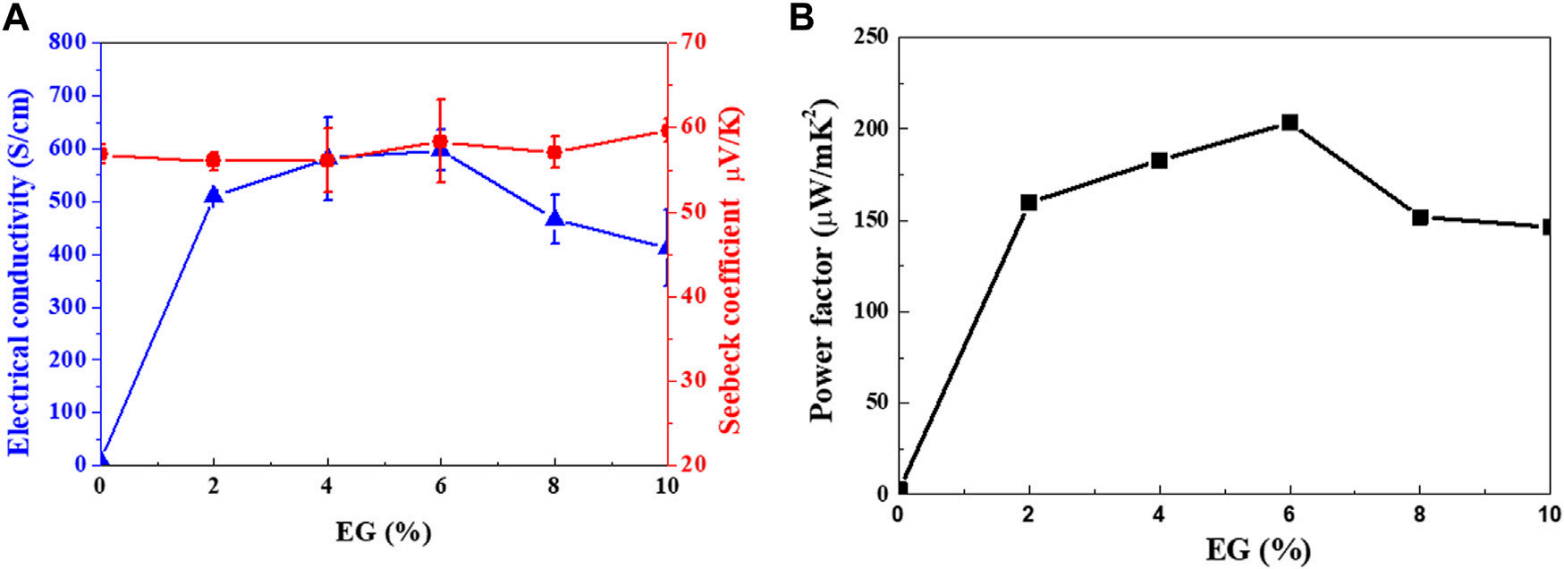
**(A)** Seebeck coefficient and electrical conductivity, and **(B)** power factor of the Se wire/PEDOT:PSS composite as functions of ethylene glycol (EG) doping concentrations.

Figure 7 shows the TE properties (*S*, *σ*, and power factor) of the Se wire/PEDOT:PSS composite with different EG doping concentrations measured at 22°C–25°C. At 0% EG, the *σ* was as low as 8.03 S/cm and significantly increased to 509, 581, and 597 S/cm at 2%, 4%, and 6%, respectively. Further increasing the amount of EG gradually decreased the *σ* to 466 S/cm (8%) and 411 S/cm (10%). The increase in *σ* with increasing EG content in PEDOT:PSS (Lim, 2013) has been previously reported. On the other hand, the *S* of the Se wire/PEDOT:PSS composite remains relatively stable between 54 and 60 μV/K. However, a slight increase is observed at higher EG content; a maximum value of 59.61 μV/K at 10% EG and a minimum of 53.86 μV/K at 4% EG is observed. Furthermore, the power factor increased with EG doping (Figure 7B). At 2% EG, it increased by 61 times compared to that at 0% EG. A maximum power factor value (203.29 μW/mK^2^) was observed at 6% EG for the PEDOT:PSS and Se wire because of the corresponding maximum electrical conductivity and unchanged *S*. Table 1 contains a summary of the properties of representative organic/inorganic composite thermoelectric. As indicated in Table 1, the prepared thermoelectric material achieves an enhancive thermoelectrical performance compared to the other samples, which is only lower than Cu_2_Se/PEDOT:PSS. However, the Cu_2_Se/PEDOT:PSS composite was synthesized through complex processes, including long chemical reaction time, hard washing, filtration process, and cold-press process sequentially.

**TABLE 1:** Comparison of the characteristics properties of organic/inorganic composite thermoelectric.

Materials	Methods	*σ* (S/cm)	*S* (μV/K)	PF (μW/mK^2^)	Ref
SiC/PEDOT:PSS	Dilution−filtration and post-treatment	3,113	20.3	128.3	Wang et al. (2018)
Cu_2_Se/PEDOT:PSS	Wet chemical process	1,047	50.8	270.3	Lu et al. (2019)
Bi_2_Te_3_/PEDOT:PSS	Hydrothermal and physical mixing methods	1,295	15.8	32.3	Cu et al. (2014)
Te nanowire/PEDOT:PSS	Wet chemical process	11	170	35	Coates et al. (2013)
Se nanowire/PEDOT:PSS	Galvanic displacement	596.76	58.3	203.29	This work

The effect of EG doping on the TE transport properties of the Se wire/PEDOT:PSS composite can be summarized as the following: 1) Compared to the undoped composite, at 2% EG, the *σ* significantly increased by more than 60 times, while the *S* remained the same. 2) As the amount of doped EG increased to 6%, *σ* and *S* simultaneously increased. 3) Above 6% EG, the *S* increased, while *σ* decreased.

In general, when *S* increases, *σ* tends to decrease; these characteristics can be correlated with the following equation:

$$\text{S}=\frac{8\pi^{2}k_{B}^{2}}{3eh^{2}}m^{*}T{\left(\frac{\pi }{3n}\right)}^{\frac{2}{3}}$$ (3)

In addition, the *σ* is expressed as follows:

σ=neμ (4)

where *k*_B_ is the Boltzmann constant, *h* is the Planck constant, *m* ^*^ is the effective mass, and *n* and *μ* are the concentration and mobility of electrons, respectively. It can be seen that *S* is inversely proportional to *n* in Eq. 3. However, for 2%–6% EG (Figure 7A), a simultaneous increase in *σ* and *S* was observed. This may be due to the carrier energy filtering effect between the organic and inorganic TE materials. Carrier energy filtering restricts unnecessary carrier movement by removing cold carriers with low carrier energy because of the difference in work function between the organic and inorganic materials; thus, even if *σ* increases, *S* cannot decrease. To confirm the optimal EG concentration, the work function of PEDOT:PSS was analyzed using UPS.

**FIGURE 8:**
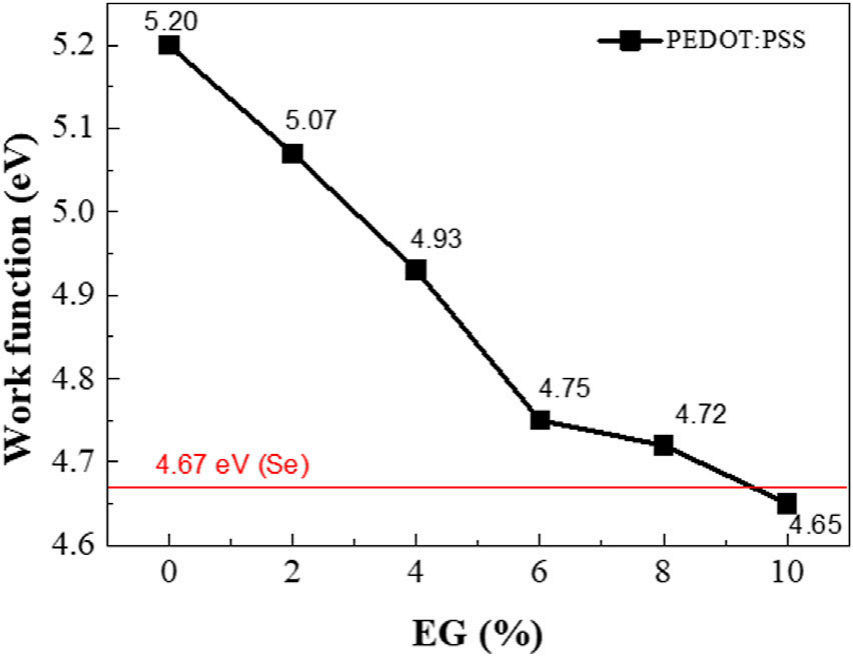
Work function of the Se wire:PEDOT:PSS composite as a function of the EG doping concentration.

It was confirmed that the work function of PEDOT:PSS decreased as a result of EG doping (Figure 8). Furthermore, the increase in the *σ* of PEDOT:PSS with increased EG doping affects the decrease in the work function.

**FIGURE 9:**
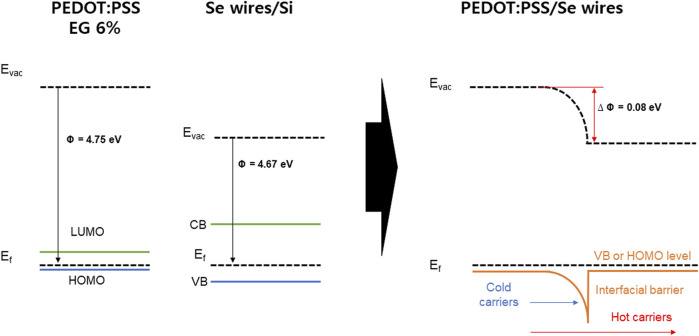
Energy diagram of the PEDOT:PSS and Se wires/Si and energy filtering effects at PEDOT:PSS/Se wires junctions.

As an example, Figure 9 shows the band bending induced at the interface between PEDOT:PSS with 6% EG (work function ∼4.75 eV) and the Se wires, which generates a possible carrier filtering effect. The work function difference between PEDOT:PSS with 6% EG and the Se wire yields a barrier height of 0.08 eV (4.75–4.67 eV). Therefore, the barrier height of the interface with the Se wires is expected to be 0.4, 0.26, and 0.08 eV for PEDOT:PSS with 2%, 4%, and 6% EG, respectively. As a result, *S* can be improved despite the increase in *σ* because of the possible carrier energy filtering effect (Song and Cai, 2017; Hu et al., 2018; Choi et al., 2016). For higher EG doping samples, the barrier height becomes very small (∼0.05 eV) and negative (−0.02 eV), which might not successfully induce a carrier filtering effect.

## Conclusion

In this study, the electrical and TE properties were measured for a GDR-grown Se wire coated with EG-doped PEDOT:PSS. For the undoped wire, the *S* and *σ* of the Se wire/PEDOT:PSS composite were 56.81 μV/Κ and 8.082 S/cm, respectively. Increasing the EG doping concentration increased the *S* and *σ* of the prepared composites, with maximum values (58.36 μV/Κ and 596.76 S/cm, respectively) observed at 6% EG. In addition, at 6% EG, the maximum value of the power factor (203.29 μW/mK^2^) was obtained. Increasing the EG concentration improved the electrical conductivity because the charge path of PEDOT:PSS was controlled; as a result, an organic/inorganic composite with improved TE properties was obtained by adding a Se wire array with a high *S*. These results suggest a source technology that can enhance the properties of composite TE materials by controlling the structure and direction.

## Data Availability

The original contributions presented in the study are included in the article/Supplementary material; further inquiries can be directed to the corresponding author.
